# Behavioural divergence during biological invasions: a study of cane toads (*Rhinella marina*) from contrasting environments in Hawai'i

**DOI:** 10.1098/rsos.180197

**Published:** 2018-04-25

**Authors:** Jodie Gruber, Gregory Brown, Martin J. Whiting, Richard Shine

**Affiliations:** 1 School of Life and Environmental Sciences, The University of Sydney, Sydney, New South Wales, Australia; 2 Department of Biological Sciences, Macquarie University, North Ryde, New South Wales, Australia

**Keywords:** alien species, *Bufo marinus*, dispersal phenotype, exploration, neophilia

## Abstract

Invasive species must deal with novel challenges, both from the alien environment and from pressures arising from range expansion *per se* (e.g. spatial sorting). Those conditions can create geographical variation in behaviour across the invaded range, as has been documented across regions of Australia invaded by cane toads; range-edge toads are more exploratory and willing to take risks than are conspecifics from the range-core. That behavioural divergence might be a response to range expansion and invasion *per se*, or to the different environments encountered. Climate differs across the cane toads' invasion range from the wet tropics of Queensland to the seasonally dry climates of northwestern Western Australia. The different thermal and hydric regimes may affect behavioural traits via phenotypic plasticity or through natural selection. We cannot tease apart the effects of range expansion versus climate in an expanding population but can do so in a site where the colonizing species was simultaneously released in all suitable areas, thus removing any subsequent phase of range expansion. Cane toads were introduced to Hawai'i in 1932; and thence to Australia in 1935. Toads were released in all major sugarcane-growing areas in Hawai'i within a 12-month period. Hence, Hawai'ian cane toads provide an opportunity to examine geographical divergence in behavioural traits in a climatically diverse region (each island has both wet and dry sides) in the absence of range expansion subsequent to release. We conducted laboratory-based behavioural trials testing exploration, risk-taking and response to novelty using field-caught toads from the wet and dry sides of two Hawai'ian islands (Oahu and Hawai'i). Toads from the dry side of Oahu had a higher propensity to take risks than did toads from the dry side of Hawai'i. Toads from Oahu were also more exploratory than were conspecifics from the island of Hawai'i. However, toads from wet versus dry climates were similar in all behaviours that we scored, suggesting that founder effects, genetic drift, or developmentally plastic responses to ecological factors other than climate may have driven behavioural divergence between islands.

## 1. Introduction

At the range-front of an ongoing invasion, vanguard individuals encounter novel ecological and evolutionary pressures that are not experienced by conspecifics from long-colonized areas [1]. There are two broad reasons why we might expect to see distinctive behavioural phenotypes in an invasive population. The first is that they are encountering novel ecological challenges (e.g. climate, habitat, predators and prey), best dealt with by specific behaviours such as a propensity to explore, engage with novel objects and take risks. The second explanation involves the process of range expansion, which may lead to higher frequencies of traits at the range-front as a result of natural selection, plasticity and non-adaptive processes such as spatial sorting (increased dispersal rates due to assortative mating of fast-dispersing individuals [2]) and surfing of deleterious mutations [3,4].

Individuals with dispersal-enhancing behavioural traits such as high levels of exploration, neophilia (an attraction to novelty) and risk-taking are predicted to be more common in invasion-front than in long-colonized populations both because they enhance an individual's ability to find food and shelter in novel environments [5–7] and because these traits increase dispersal rates [8,9]. In keeping with this prediction, dispersal-enhancing behavioural traits are associated with range expansion and invasion success in several species (table 1). A key question in invasion biology is whether variation in behavioural traits during an invasion is a consequence of encountering novel environments, such as new climatic conditions, or due to the pressures imposed by range expansion? To answer this question, we need a study system with multiple invasive populations that differ in whether or not they are undergoing range expansion.

**Table 1.** Examples of variation in dispersal-enhancing behavioural traits among invasion-front and long-colonized populations across an invasion range.

behavioural traits	species	pattern of divergence	reference
boldness (emergence/risk-taking) exploration	round goby (*Neogobius melanostomus*)	individuals from the range-edge emerged sooner and moved further and faster than did conspecifics from long-established populations	Groen *et al*. [10], Myles-Gonzalez *et al*. [11]
boldness sociality	mosquitofish (*Gambusia affinis*)	bolder and less social individuals drive dispersal and range expansion	Cote *et al*. [5]
aggression	western bluebird (*Sialia Mexicana*)	males from invasion-front populations were more aggressive than were conspecific males from long-established populations	Duckworth & Badyaev [12]
exploration	dark-eyed junco (*Junco hyemalis*)	individuals that recently invaded novel urban habitats were more exploratory than were conspecifics from wildland populations	Atwell *et al*. [13]
neophilia exploration	house sparrow (*Passer domesticus*)	invasion-front individuals were more exploratory and likely to eat novel foods than were conspecifics from core populations	Liebl & Martin [14,15]

The cane toad (*Rhinella marina* [Linnaeus 1758]) is a notoriously successful invasive species [16]. Originally from Central and South America, cane toads were introduced to the Caribbean in an attempt to control pests in sugar cane plantations before being introduced to Hawai'i in 1932 [16,17]. Cane toads from the Hawai'ian population were introduced to Australia in 1935 [18,19]. The ongoing cane toad invasion in Australia has been accompanied by divergence in behavioural traits across the invasion range. Invasion-front toads are more exploratory and more willing to take risks than are conspecifics from long-colonized populations [20]. Such behavioural divergence across the cane toads' Australian invasion range may be related to range expansion, or to other factors such as variation in climate between the range-edge and the range-core. Cane toads from long-colonized populations in north-eastern Australia experience a monsoonal climate with a warm wet season followed by a cooler dry season, whereas cane toads at the invasion-front in northwestern Australia experience long periods of intense heat and aridity [21]. Such climatic differences could plausibly lead to divergence in cane toad behaviour via adaptation (if there is a differential fitness benefit of dispersal-enhancing behavioural traits across the range) or through plastic behavioural responses to changing climates.

Climatic factors such as temperature and precipitation can have profound effects on an organism's physiology, morphology, life-history and fitness [22]. Geographical variation in climate has been linked to divergence in phenotypic traits across species ranges in many taxa. For example, cold temperatures lead to larger body size (Bergmann's Rule, [23,24]) in birds [25], insects [23,26] and chelonian reptiles [27]. And water uptake in two species of anuran, *Rhinella humboldti* and *Leptodactylus fuscus*, is slower in individuals from wet versus dry environments [28].

Climatic factors may also affect behavioural traits. For example, individuals may respond to sub-optimal temperatures or precipitation by behavioural thermoregulation, reducing energetically costly dispersal behaviours [23,26,29,30]. Temperature and rainfall also may influence dispersal-related behaviours by constraining the available habitat and thus, influencing an individual's ability to disperse out of their home range [28]. For example, in dry regions, dispersal-enhancing traits such as exploration, risk-taking and neophilia may be selected against and philopatry to known water and food resources may be favoured [31]. In contrast, in areas where rainfall is high and aseasonal, dispersal-enhancing behaviours may be favoured as the risks of desiccation with dispersal are low and dispersing individuals may benefit from accessing new resource patches [32].

Amphibians are acutely sensitive to the temperature and humidity of their local climate because of the challenges they face maintaining optimal body temperatures and water balance with their permeable skin, and because they rely on water bodies for survival and reproduction [29,33]. Several anuran species exhibit behavioural adaptations to climate, particularly in response to arid conditions. For example, specialized behaviours allowing individuals to seek out and remain within close proximity to humid refugia, are common in anurans [34]. Cane toads from the seasonally arid areas of their Australian invasion range exhibit behavioural plasticity in response to climatic conditions; exhibiting higher philopatry to water sources in dry versus wet conditions [29,35]. There is also evidence of a link between climate and divergence in behavioural traits across the invasion range; toads from the arid range-edge maintain higher locomotor performance under desiccating conditions than do conspecifics from the wetter range-core [36]. Hence, the geographical divergence of dispersal-related behaviours such as exploration, risk-taking and neophilia evident in cane toads across their Australian invasion range [20] may be driven by responses to the profoundly different climates experienced by individuals inhabiting the range-edge versus the range-core. Because the cane toads' Australian invasion is still expanding, it is difficult to distinguish the effects of climate from those of range expansion. To disentangle the effects of range expansion versus climate on behavioural divergence in cane toads, we need an invasive population that occurs across geographically divergent climates, but that has not undergone a period of extended range expansion.

In Hawai'i, cane toads were distributed to all sugarcane-growing areas on the main islands within a brief period (greater than 100 000 individuals released from 1933 to 1934; [17]). Subsequent dispersal has been minimal, due to the arid volcanic mountains that separate the wet and dry sides of the Hawai'ian islands [16,19,37]. In contrast, cane toads were introduced (from Hawai'i) to Australia at about the same time (1935) but in only one area (along the northeast coast), and have since spread out more than 3000 km to the west [38]. Like Australia, the Hawai'ian islands exhibit profound spatial heterogeneity in climate; but in Hawai'i, that heterogeneity involves the contrast between a wet (windward) and dry (leeward) side on each island [39]. Here, we used standardized laboratory-based behavioural assays to test dispersal-related behaviour of toads from wet and dry areas on two Hawai'ian islands, allowing us to investigate if behavioural divergence is driven by climate in this species.

The Hawai'ian landscape is rugged with porous volcanic soil. Rainfall differs between the eastern windward (wet) and western leeward (dry) sides of the Hawai'ian islands; wet sides have high levels of aseasonal rainfall, while rain-shadows lead to extreme aridity on the dry side of each island [37,39]. Cane toads that inhabit the dry side of each island are mostly confined to anthropogenically created oases within an otherwise arid and hostile landscape. In contrast, on the wet side of each island where rainfall is frequent, the landscape is more homogeneous and there is ground moisture and vegetation cover, enabling toads to disperse more freely [37,39]. Hence, toads on the dry side of each island may be no more water-stressed than are conspecifics in wet areas (as they inhabit well-watered areas such as parks and gardens), but dispersal of dry-side toads may be restricted by the lack of rainfall and the arid matrix. In keeping with this prediction, toads are widely dispersed across the wet side of each island, but are restricted to well-watered parks and golf courses on the dry side [37].

Dispersal-enhancing behavioural traits may be selected against in individuals inhabiting a small habitat patch that provides resources such as food, water and shelter in a low-rainfall area, within an otherwise arid matrix [40]. This is because the costs of dispersal outweigh the benefits (such as decreased competition and access to new resource patches) [41]. Behavioural traits that enhance exploration and dispersal out of the home range may be particularly costly for cane toads inhabiting the dry side of each Hawai'ian island because in the surrounding matrix food is scarce and standing water-bodies for breeding and rehydration are rare [37]. Hence, we predict that toads inhabiting the dry side of each island will exhibit low levels of exploration, risk-taking and neophilic behavioural traits due to the concentration of essential resources and the costs associated with dispersal from these human-created oases. In contrast, we predict that toads on the wet side of each island will exhibit high levels of exploration, risk-taking and neophilic behavioural traits as the high and aseasonal rainfall allows dispersal across the landscape, providing access to new resource patches.

## 2. Material and methods

### 2.1. Study animals and maintenance

In 2015, we collected 60 male and 60 female cane toads (*Rhinella marina*) at night and by hand from four populations (30 toads/population): the wet (windward) side and the dry (leeward) side of Oahu, and the wet (windward) side and dry (leeward) side of Hawai'i (henceforth referred to as the Big Island, to avoid confusion). Mean annual rainfall averages are between 3550 and 7850 mm at the ‘wet’ sites both on Oahu and the Big Island, and between 204 and 2750 mm in the ‘dry’ sites of both islands (average rainfall for the 30-year period, 1978–2007 [42]). Fifteen toads of each sex were collected from each of four sample populations (Oahu dry-side, Oahu wet-side, Big Island dry-side, Big Island wet-side) and toads were collected from three sub-sample sites (mostly constituting separate parks and golf courses) within each population. Toads greater than 90 mm snout–urostyle length (SUL) were classified as adults and their sex was determined by the absence of nuptial pads on the fore-limbs and lack of a ‘release call’ when held (only males have nuptial pads and are able to make release calls in this species [43]). Toads were weighed to the nearest 0.1 g on a digital scale, and measured (SUL) to the nearest 0.01 mm using digital calipers. They were transported in moist calico bags to an animal-holding facility near the town of Hilo on the Big Island, where they were immediately released into their housing tubs and provided with water.

Toads were housed in 70 l plastic tubs (measuring 645 mm × 413 mm × 397 mm) in groups of three to four individuals per tub. Each tub had a substrate of wood-chip bedding and contained two opaque plastic shelters, and a large shallow water dish. Toads were fed store-bought crickets (*Gryllus bimaculatus*) or mealworms (*Tenebrio molitor*) every 2 days and water was provided ad libitum. Because adult toads are most active at night [16], room lights were activated from 03.30 to 15.30 h each day, to allow behavioural trials to be carried out in the dark phase between 15.30 and 22.00 h. Toads showed no signs of stress or illness and ate well, either maintaining or gaining weight during their time in captivity.

### 2.2. General methods

Each toad was tested in three different behavioural trials: (i) exploration, (ii) risk-taking (emergence from a shelter into the test arena), and (iii) neophilia (response to a novel stimulus). Trials ran for 30 min and four trials with one toad per trial arena were conducted simultaneously in each trial round. It took five trial rounds (of four toads running simultaneously) to run 20 toads in 1 day, and 6 days to run all animals through each trial type. Toads were randomly selected from each population and sex group, with the proviso that an equal number of toads from each population and each sex were represented in each trial round (e.g. in any one round, an equal number of randomly selected males and females from the wet and dry Oahu or wet and dry Big Island were represented). Each toad had 2 days of rest between trial types (while the other sets of 20 toads were assayed). Toads from the two islands (Oahu and the Big Island) were assayed consecutively rather than simultaneously due to logistical and space constraints.

**Figure 1.**
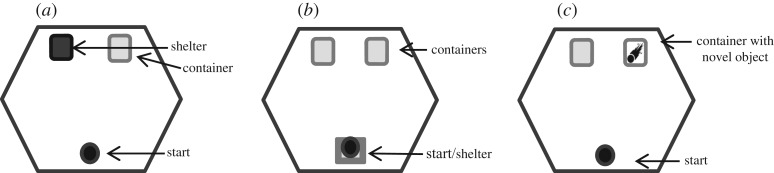
Layout of arenas for trials testing for divergence in dispersal-related behaviours in Hawai'ian cane toads: (*a*) exploratory trial—arena with an empty container and accessible shelter opposite the start point of the toad (depicted by a circle), (*b*) risk-taking (emergence) trial—arena with a toad inside the start shelter and two empty containers opposite the toad start point, (*c*) novel object trial—arena with a toad in the starting position and two containers at the opposite end of the arena; one container is empty and the other contains a moving novel object.

Trial arenas were large (120 × 120 × 83 cm) hexagonal pens made from waterproof fabric with an open top to allow filming from above (figure 1). All trials were filmed using CCTV cameras under low-level red light and we scored videos using Ethovision XT10 behavioural analysis software (ensuring that blind methods were used to score the videos). Researchers left the room at the start of trials to minimize interference. The PVC substrates (and shelters, etc.) of each arena were wiped down with diluted ethanol before each trial to eliminate scent from previous trials. We measured the arena substrate temperature before each trial (mean: 28 ± 2 s.d. °C). At the start of a trial, toads were placed under shelters for five minutes before the refuge was removed. During ‘neophilia’ trials, toads were presented with a novel object (a blue and red silicone squid-mimic fishing lure that moved in a bobbing motion every 2 s to simulate a novel prey item) inside a clear plastic container. Because toads may have approached and settled next to the container to seek a hiding place (rather than out of interest in the novel object), we included an empty container in all trials to disambiguate hiding behaviour from interest in novel object (for consistency in the layout of the arena, empty containers were also included in exploration and emergence trials; figure 1).

### 2.3. Behavioural trial protocols

#### 2.3.1. Exploratory trial

To test exploration and space-use in a novel environment [9,44,45], we measured time spent moving and rate of movement [46,47]. Toads were given 5 min rest under a shelter before the shelter was removed and trials began. An empty container and a shelter were placed at one end of the arena (figure 1). The shelter gave toads the option to hide during the trial, allowing us to distinguish bold exploratory behaviour from fear-driven escape behaviour (see [48]).

#### 2.3.2. Emergence trial

To score risk-taking behaviour, we recorded whether or not a toad emerged from its shelter, and latency to emerge (s). Two empty containers were placed at the opposite end of the arena to the shelter (figure 1). The entrance to the shelter was initially covered, and toads were given 5 min rest under the shelter before the entrance cover was removed and trials began. A shorter latency to emerge from the shelter indicates higher risk-taking propensity in animals generally [44,49,50]. We defined emergence into a novel environment from a safe shelter as a measure of risk-taking, whereas exploration behaviour is defined as space-use in a novel environment [45,51].

#### 2.3.3. Novel object trial

To test an individual's attraction or aversion to a moving novel object (intended to simulate a moving novel prey item), we added a red and blue silicone fishing lure (20 × 10 mm, mimicking a squid) driven by a small motor to move up and down in a fixed position every 2 s (figure 1). The novel object was inside a container to prevent the toad from consuming the plastic lure. Toads were given 5 min rest under a shelter, which was placed at the opposite end of the arena to the novel object, before the shelter was removed and trials began. We measured whether or not the toad approached the novel object, the latency to approach the novel object, and the time the toad spent within 2 cm of the novel object.

### 2.4. Statistical analysis

We used general linear models to analyse the effects of island (Oahu–Big Island), climate (wet–dry) and their interaction on behavioural traits. The potentially confounding factors sex, mass (g), arena and trial number were included as main effects in all models. We ran separate models for all behavioural traits. Prior to building our final model, we ran investigatory models comparing the three sub-populations within each main population (Oahu dry, Oahu wet, Big Island dry, Big Island wet) to look for variation in behavioural traits. We found that sub-populations did not differ significantly in any behavioural traits within each main population, thus we grouped sub-populations together to increase the power of our final models. We used Tukey's post hoc tests to run pairwise comparisons to interpret the nature of trait variation as appropriate. We also used Spearman's *ρ* correlation analysis to examine pairwise correlations between behavioural traits from different trial types. All data were checked for normality and homoscedasticity and log-transformed to meet these assumptions as required. All data were analysed using the ‘car’ package [52] in R [53]. We used generalized linear models (‘glm’ function in R) with a binomial logit link function to compare whether or not toads emerged during risk-taking trials and whether toads approached within 2 cm of the novel object; during neophilia trials.

The behavioural traits measured for each trial type were: (i) total time spent moving, and rate of movement (as quantified by the residual scores from a general linear regression of total distance moved against total time spent moving; exploratory trials); (ii) emergence (binomial, whether or not individuals emerged during trials) and latency to emerge (emergence trials); and (iii) whether or not toads approached to within 2 cm of the novel object (binomial), latency to approach and time spent within 2 cm of the novel object; novel object trials).

## 3. Results

**Figure 2.**
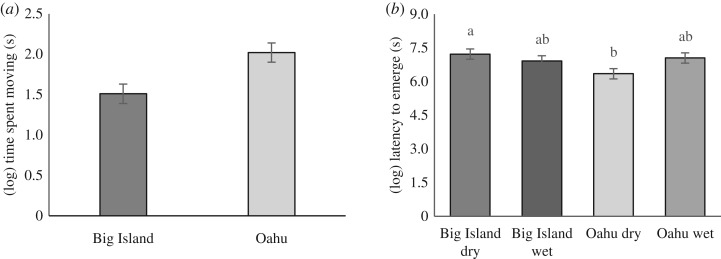
(*a*) Time spent moving (s) during exploration trials and (*b*) latency to emerge (s) during risk-taking (emergence) trials testing behavioural divergence of these traits in cane toads from wet- and dry-climate populations of Oahu and the Big Island, Hawai'i.

Toads from Oahu spent more time moving during exploration trials than did conspecifics from the Big Island (table 2, figure 2*a*). We found no interaction effect between island and climate, nor an effect of any other factors on the time toads spent moving during exploration trials (table 2). Similarly, there was no effect of an interaction between island and climate, island and climate alone, nor sex, mass or trial number on the rate of toad movement (table 2). There was an effect of arena on movement rate; toads trialled in arena number one had a higher rate of movement than did toads in any of the other three arenas (table 2). However, this significant effect occurred only for this behavioural measure.

**Table 2.** Effects of island (Oahu versus Big Island), climate (wet versus dry), their interaction (island*climate) and the potentially confounding factors mass, sex, arena and trial number on behavioural traits during exploration (time spent moving, rate of movement), risk-taking (proportion to emerge and latency to emerge), and neophilia (proportion to approach novel object and time spent with novel object) trials. Results for main effects are based on analyses after exclusion of non-significant interaction terms. Statistically significant values (*p* ≤ 0.05) are highlighted in bold text.

behavioural trial	variable	island	climate	island*climate	sex	mass (g)	arena #	trial #
exploration	time spent moving (s)	***F*_1,117_** = **10**.**08** ***p*** = **0**.**002**	*F*_1,117_ = 2.66 *p* = 0.11	*F*_1,117_ = 0.0001 *p* = 0.99	*F*_1,117_ = 0.13 *p* = 0.71	*F*_1,117_ = 1.99 *p* = 0.16	*F*_1,117_ = 2.38 *p* = 0.13	*F*_1,117_ = 2.69 *p* = 0.10
	movement rate	*F*_1,117_ = 1.33 *p* = 0.25	*F*_1,117_ = 0.22 *p* = 0.64	*F*_1,117_ = 0.03 *p* = 0.87	*F*_1,117_ = 0.54 *p* = 0.46	*F*_1,117_ = 1.31 *p* = 0.25	***F*_1,117_** = **8**.**59** ***p*** = **0**.**004**	*F*_1,117_ = 0.0009 *p* = 0.98
risk-taking	proportion to emerge	*χ*^2^ = 0.02 *p* = 0.90	*χ*^2^ = 2.67 *p* = 0.10	*χ*^2^ = 2.14 *p* = 0.14	*χ*^2^ = 3.57 *p* = 0.06	*χ*^2^ = 0.12 *p* = 0.72	*χ*^2^ = 2.01 *p* = 0.16	*χ*^2^ = 0.29 *p* = 0.59
	emergence latency (s)	*F*_1,117_ = 3.70 *p* = 0.06	*F*_1,117_ = 0.64 *p* = 0.43	***F*_1,117_** = **6**.**11** ***p*** = **0**.**02**	*F*_1,117_ = 0.04 *p* = 0.84	*F*_1,117_ = 2.42 *p* = 0.12	*F*_1,117_ = 0.008 *p* = 0.93	*F*_1,117_ = 3.16 *p* = 0.08
neophilia	proportion to approach novel object	*χ*^2^ = 0.005 *p* = 0.94	*χ*^2^ = 0.02 *p* = 0.87	*χ*^2^ = 0.006 *p* = 0.94	*χ*^2^ = 0.64 *p* = 0.42	*χ*^2^ = 0.21 *p* = 0.65	*χ*^2^ = 0.20 *p* = 0.65	*χ*^2^ = 2.11 *p* = 0.15
	time spent with novel object (s)	*F*_1,117_ = 0.74 *p* = 0.39	*F*_1,117_ = 0.003 *p* = 0.96	*F*_1,117_ = 2.20 *p* = 0.14	*F*_1,117_ = 2.53 *p* = 0.11	*F*_1,117_ = 1.14 *p* = 0.29	*F*_1,117_ = 0.26 *p* = 0.61	*F*_1,117_ = 1.20 *p* = 0.28

We found an interaction effect of island and climate on the latency of toads to emerge from the shelter during risk-taking trials; toads from the dry side of the Big Island took significantly longer to emerge than did toads from the dry side of Oahu (table 2, figure 2*b*). During risk-taking trials, similar proportions of Oahu and Big Island toads from both wet and dry climates emerged and had similar emergence latencies (table 2). Toads from each island and climate type also responded in a similar way to the novel object during neophilia trials (table 2). Other factors such as sex, mass, arena and trial number had no significant effect on any behavioural traits during risk-taking or neophilia trials (table 2). Spearman's *ρ* analysis showed significant correlations between only two of the six pairwise correlations of behavioural variables measured from different trial types (exploration, risk-taking and neophilia), suggesting that the different trial types largely measured different components of behaviour (table 3). The two significant correlations were between time spent moving in the exploration trial and time spent with the novel object during the neophilia trial, and between time spent moving and rate of movement (both measured during the exploration trial; this result may reflect the fact that we calculated rate of movement using residuals from a regression of time spent moving and distance moved: table 3).

**Table 3.** Spearman's *ρ* pairwise comparisons between behavioural variables measured across different trial types testing for divergence in exploration, risk-taking and neophilic behaviours in cane toads (*Rhinella marina*) from wet versus dry climates on Oahu and the Big Island, Hawai'i. Statistically significant values (*p* ≤ 0.05) are highlighted in bold text.

pairwise correlations of behavioural variables	*ρ*	*p*
time spent moving, movement rate	0.22	**0**.**02**
time spent moving—latency to emerge	−0.16	0.08
latency to emerge, movement rate	0.08	0.38
time spent moving, time spent with novel object	0.29	**0**.**002**
latency to emerge, time spent with novel object	0.11	0.25
time spent with novel object, movement rate	−0.02	0.80

## 4. Discussion

We found that the brief period of separation (slightly more than 80 years) of the four populations of cane toads that we sampled has been accompanied by divergence in behavioural traits. In our trials, toads from the dry side of Oahu were bolder; that is, they were quicker to emerge from a shelter than were conspecifics from the dry side of the Big Island. Toads from both wet and dry sides of Oahu were also more exploratory (spent more time moving in a novel environment) than were conspecifics from the Big Island. Contrary to our prediction, behavioural traits of cane toads did not differ between populations from wet versus dry climates.

Why did behaviour not differ between toads from wet versus dry climates? There is a marked disparity in hydric regimes between the wet (windward) and dry (leeward) sides of each island [39,54]. For example, mean annual rainfall averages are between 3550 and 7850 mm on the ‘wet’ sites both on Oahu and the Big Island, and between 204 and 2750 mm on the ‘dry’ sites of both islands [42]. Frequent rainfall on the wet sides of both islands may allow toads to disperse through the landscape between favourable habitat patches, whereas such dispersal is unlikely on the dry side of each island [37]. Hence, we predicted that behavioural divergence among populations would reflect this dichotomy in hydric regimes, not through physiological water-stress *per se* (as dry-side toads often inhabit well-watered human-created landscapes) but because the hydric environment restricts toad movement and dispersal on the dry sides of the islands.

The hypothesis that toad behaviour evolves in response to hydric regimes predicts that toads from the leeward side of each island should be more similar to each other than they are to toads from windward sites on the same island (and vice versa). Our data did not support this prediction. First, the behavioural divergence we saw in Hawai'ian toads was in exploration behaviour between islands and in risk-taking behaviour between the dry sides of each island, not between ‘wet’ and ‘dry’ habitats (figure 2). Clearly, then, effects or adaptations related to water availability cannot explain the behavioural divergence that has accumulated in invasive populations of cane toads over the last 80 years.

The lack of significant effects of climate on behavioural traits may be due to similarities in the local environment in the areas toads inhabit on the wet and dry sides of each island. Cane toads in Hawai'i mostly inhabit well-watered anthropogenically created landscapes such as parks and golf courses, hence toads from both wet and dry climates experience similar local hydric regimes [37]. We predicted that toads on the dry side of the islands would be less exploratory, risk-taking and neophilic because of the costs of dispersal and movement restrictions enforced by the arid landscape surrounding these human-tended home ranges. However, toads may not need to venture beyond their human-created oases in order to access resources such as food, mates and shelter, favouring similar behaviours in all locations.

Although we found no effect of wet versus dry climates on toad behaviour, we did find a difference in exploration behaviour between the two islands. Toads from Oahu spent more time moving than did Big Island conspecifics. Risk-taking behaviour also differed between toads from the dry sides of each island; Oahu toads were quicker to emerge from a shelter than were Big Island toads. The dry side of Oahu has a higher average annual rainfall than does the dry side of the Big Island, while average annual rainfall on the wet side of Oahu is lower than on the wet side of the Big Island (dry-side average annual rainfall: Oahu 499 mm, Big Island 234 mm; wet-side average annual rainfall: Oahu 2473 mm, Big Island 3500 mm; [42]). The higher rainfall on the dry side of Oahu may provide more windows of opportunity for toads to explore beyond their home ranges, compared to those of toads inhabiting lower rainfall areas on the dry side of the Big Island. However, we doubt that variation in climate drives behavioural divergence as we did not find significant behavioural differences between populations with the most acute opposites of climate: that is, populations from the wet versus the dry sides of each island. The most parsimonious explanation for between-island differences in behaviour may be genetic drift. That explanation, however, is weakened by the fact that founding populations at each site contained large numbers of individuals (reducing stochastic effects on allele frequencies [55]) and the putative lack of gene flow between populations on the ‘wet’ and ‘dry’ sides of each island. If divergence was due to genetic drift, we would not expect parallel evolution in the two isolated sides of each island. All we can suggest is that some as-yet-undocumented ecological difference between Oahu and the Big Island (such as prey availability, habitat complexity or predation pressure) has generated differences in toad behaviour, either as an adaptation or as a phenotypically plastic response.

Alternatively, the geographical divergence in exploration behaviour (between cane toads from Oahu versus the Big Island) and risk-taking behaviour (between cane toads from the dry side of each island) might be genetically based, despite the short time-frame of their isolation. Over the same period, and despite a continuous distribution, populations of cane toads across tropical Australia have diverged in many phenotypic traits; and the same divergences are seen in offspring raised in ‘common garden’ experiments [56–59]. Cane toads are, therefore, capable of evolving heritable differences within the timespan over which populations in the two Hawai'ian islands have been isolated from each other.

Further research could usefully explore the ecological context of cane toad ontogeny at a range of Hawai'ian sites, to clarify the as-yet-unexplained pattern of consistency in some behavioural traits within but not between islands. It would also be informative to compare factors that may affect behaviour such as resource availability, population densities and competition between wet and dry sites; and to study the mechanistic basis for inter-island divergence in exploration and risk-taking traits by raising offspring in common garden experiments (as has been done for Australian populations [58–60]). Even without answering these further questions, however, our data show that 80 years of genetic isolation has been accompanied by significant divergence of exploration and risk-taking behavioural traits between, but not within, the two Hawai'ian islands that we studied. Our results reinforce the ability of invasive populations to diverge rapidly, even in the absence of evolutionary pressures associated with prolonged range expansion.

## Supplementary Material

Dataset for MS title: Behavioural divergence during biological invasions: a study of cane toads (Rhinella marina) from contrasting environments in Hawai'i

## Data accessibility

The dataset supporting this article has been uploaded as part of the electronic supplementary material.
